# The Contagiousness of Pulmonary Consumption as to the Married

**Published:** 1883-04

**Authors:** I. Burney Yeo


### THE CONTAGIOUSNESS OF PULMONARY CONSUMPTION AS TO THE MARRIED.

I. Burney Yeo, M. D.

It seems certain that the communication of consumption from wife to husband, even among the class in which the conditions of life favor to the utmost the communication of contagious disease, is very rare; while it would seem that communication (assuming, for the sake of argument, the disease really was communicated) from husband to wife is more frequent.

Dr. Hermann Weber brought the subject of the communicability of consumption from husband to wife before the Clinical Society, and in his paper states that he possesses the history of "sixty-eight persons, male and female, who, with a more or less pronounced consumptive taint, have married healthy partners. One or several of the partners of ten out of these sixty-eight cases became consumptive. The question, however," he says, "takes a different aspect if the originally tainted husbands and wives are considered separately. Of the sixty-eight persons, thirty-nine were husbands, twenty-nine wives. Only one of the husbands of the twenty-nine wives became diseased, while the wives of nine out of the thirty-nine husbands became affected. These nine husbands lost eighteen wives, viz.: one lost four wives, one lost three, four others lost two each, and three only one each."

One of Dr. H. Weber's cases is certainly very remarkable. A young man, who had lost his mother, two brothers, and a sister of phthisis, and who himself had twice had hemorrhage from the lungs, had quite recovered, and married at twenty-seven, being then perfectly well. His first wife was in good health and came of a healthy family. She died of consumption after her third confinement. The man shortly married again, an "apparently healthy woman," and this second wife, after a year of married life, died of "galloping consumption." He again married a healthy young woman of twenty-five, belonging to "an exceptionally healthy family." During her second pregnancy she developed symptoms of phthisis, which run a rapid course, and ended fatally in about eight months. Undaunted, this man married a fourth wife, a perfectly healthy woman of twenty-three, of healthy antecedents. Three months after her first confinement she began to show symptoms of phthisis, and, notwithstanding two sea-voyages, died after nine months, with tubercle in liver, spleen, and in the intestines, as well as in the lungs. Though the husband of these four wives, who was a sailor, remained in apparently good health, physical examination revealed the existence of morbid changes about the apex of left lung. It is possible that the life at sea kept his disease in abeyance, for, when he had to lie by on account of a severe fracture, the disease became active, and he died of consumption within two years.

In Dr. Weber's second case, three wives in succession of a consumptive husband died of phthisis, the husband ultimately dying of that disease. The disease in the wives appeared during pregnancy or soon after delivery. The same story is repeated, with but little variation, except as to the number of wives, in Dr. Weber's seven other cases. Altogether he had observed thirty-nine diseased husbands, and the wives of nine of them became consumptive after marriage; but, as several of the diseased husbands married repeatedly, it would appear that, out of fifty-one such marriages, eighteen wives became consumptive after marriage. As a set-off against this, out of twenty-nine marriages between consumptive wives and healthy husbands, only one husband became consumptive. Another noteworthy observation of Dr. Weber's was, that in the infected wives the disease manifested itself in an unusually active, florid form and ran an unusually rapid course, while in the husbands it was chronic, stationary, and apyretic. The fact of the onset of the disease following or occurring in connection with impregnation and utero-gestation, as well as the fact of the immensely greater proportion of wives infected by husbands compared with that of husbands infected by wives, naturally provoked the suggestion that the latter became infected through impregnation and from the fœtus *in utero,* just as constitutional syphilis is conveyed from husband to wife. But there is another hypothesis equally tenable, and perhaps more in accordance with modern research, which is that during the puerperal state the female constitution is peculiarly prone to the reception and cultivation of the germs of infective disease, and, assuming for the sake of argument that the tubercle is propagated through the agency of an infective organism, the puerperal state may supply one of the conditions (such, for example, as we could conceive an increased body-temperature to supply) necessary for its cultivation and spread. —*British Medical Journal.*

